# Real-world safety evaluation of musculoskeletal adverse events associated with Korean pediatric fluoroquinolone use: a nationwide longitudinal retrospective cohort study

**DOI:** 10.1038/s41598-019-56815-y

**Published:** 2019-12-27

**Authors:** Yoonhye Kim, Minwoo Paik, Chanjoo Khan, Yae-Jean Kim, EunYoung Kim

**Affiliations:** 1 Clinical Data Analysis, Evidence based clinical research Lab., Departments of Health Science & Clinical Pharmacy, College of Pharmacy, Chung-Ang University, Seoul, 06974 Republic of Korea; 2 Department of Pharmaceutical Industry, Chung-Ang University, Seoul, 06974 Republic of Korea; 3 Department of Paediatrics, Samsung Medical Centre, Sungkyunkwan University School of Medicine, Seoul, 06351 Republic of Korea

**Keywords:** Bacterial infection, Paediatric research, Risk factors

## Abstract

Though the pediatric use of fluoroquinolones (FQs) is limited for musculoskeletal safety concerns, the clinical usefulness still exists. This study examined the association between FQs and musculoskeletal adverse events (AEs) as well as the possible risk factors associated with the pediatric FQs uses. This population-based, longitudinal, retrospective study was conducted using Korean National Sample Cohort database originating between 2002 and 2015. An FQ-treated pediatric cohort (<18 years old) was compared to a control treated with amoxicillin. Propensity score matching (PSM) and a Cox proportional hazard model was used to estimate the hazard ratio (HR) for a diagnosis of musculoskeletal AEs within 60 days of the first prescription. Among one million participants, total of 15,706 and 147,840 children were eligible for the FQ and amoxicillin cohorts, respectively. The PSM cohorts showed a slightly increased risk of musculoskeletal AEs after FQ treatment (HR, 1.19; 95% confidence interval, 1.01–1.40; *p* = 0.042). This association was stronger in males, older patients, and some FQs users. This study indicates that pediatric FQ use is associated with a risk of musculoskeletal AEs and that FQ use should be carefully monitored in groups with certain risk factors. Well-designed pragmatic trials could be expected to clarify these issues.

## Introduction

Fluoroquinolones (FQs) are antibiotics that act by inhibiting bacterial topoisomerases II and IV, which are essential for DNA replication. FQs are some of the most frequently used antibiotics in adult patients because of their broad-spectrum coverage, excellent tissue penetration, and relatively good safety profile^1^. But FQ use is limited in pediatrics because articular cartilage toxicity in weight-bearing joints has occurred in juvenile animal experiments^2,3^. However, the results from previous studies regarding FQ-induced musculoskeletal adverse events (AEs) in pediatrics are controversial^4–8^. The emergence of multidrug-resistant pathogens and the failure of first-line treatments have increased the need for special antibiotics like FQs^9^. Also, FQ-associated musculoskeletal AEs in children are known to be reversible and transient^6,10^. A recent study confirmed that FQ prescriptions to pediatric patients increased between 2006 and 2013^11^. The US Food and Drug Administration (FDA) licensed two FQs, ciprofloxacin and levofloxacin, for pediatric indications including inhalational anthrax, complicated urinary tract infections, and pyelonephritis^12,13^. However, FQs are still used off-label in many countries including South Korea. In a recent study, non-negligible quantities of FQs are prescribed off-label despite the drug utilization review system to control the pediatric use of FQs^14,15^. Considering the gap between the real-world use of FQs in pediatrics and musculoskeletal toxicity concerns, discovering an association between FQ use and musculoskeletal AEs in pediatric patients may have important clinical implications. However, there are few randomized controlled trials (RCTs) that have prospectively conducted to detect FQ-induced musculoskeletal diseases with an incidence of approximately 2%^10,16^. Several studies have investigated FQ-associated AEs in adults via observational studies, but very few studies have been conducted in children. In addition, no study has explored the effects of potential risk factors for musculoskeletal AEs related to pediatric FQ use. Therefore, the goals of this population-based, retrospective, longitudinal cohort study were: (1) to investigate the association between pediatric FQ use and musculoskeletal disorders and compare it with that of amoxicillin use, and (2) to identify possible risk factors that may modify this association, using a nationwide database from South Korea.

## Results

### Baseline characteristics of patients in study cohorts

**Figure 1:**
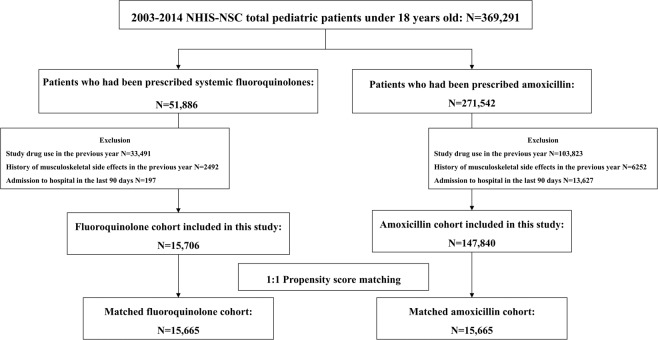
Process of selection of the study population.

Figure 1 outlines the process used to select the study population. In the National Health Insurance Service–National Sample Cohort (NHIS–NSC), the number of pediatric patients less than 18 years old from January 2003 to December 2014 was 369,291. Of these, 51,886 were prescribed systemic FQs, and 271,542 patients were prescribed amoxicillin. After exclusion, 15,706 patients were eligible for inclusion in the FQ cohort, and 147,840 patients were included in the amoxicillin cohorts. Ofloxacin was the most frequently used FQ (64.75%) followed by ciprofloxacin (13.23%), norfloxacin (11.26%), and levofloxacin (9.51%). The 1:1 propensity score-matched cohort consisted of 15,665 patients.

Table 1 lists the demographic and clinical characteristics of patients in the initial and propensity score-matched cohorts. The mean age at first prescription was significantly higher in the FQ cohort than in the amoxicillin cohort (13.46 ± 3.08 vs 3.66 ± 5.13 years, p < 0.0001). However, after propensity score matching, baseline characteristics between the FQ and amoxicillin cohorts were well-balanced.

**Table 1:** Demographic and clinical characteristics of patients in the initial and 1:1 propensity score-matched cohorts.

Patient characteristics	Initial cohort			Propensity score-matched cohort		
	FQ (n = 15 706)	Amoxicillin (n = 147 840)	Standardized difference	FQ (n = 15 665)	Amoxicillin (n = 15 665)	Standardised difference
Male gender	7832 (49.87)	75 501 (51.07)	0.02	7817 (49.90)	7904 (50.46)	0.01
Age^a^, years						
≤5	254 (1.62)	111 925 (75.71)	2.34	254 (1.62)	270 (1.72)	0.01
6–10	2466 (15.70)	12 716 (8.60)	0.22	2466 (15.74)	2418 (15.44)	0.01
11–17	12 986 (82.68)	23 199 (15.69)	80	12 945 (82.64)	12 977 (82.84)	0.01
Mean (SD)	13.46 (3.08)	3.66 (5.13)	—	13.45 (3.08)	13.49 (3.10)	—
Comorbidities						
Asthma	2846 (18.12)	22 888 (15.48)	0.07	2813 (17.96)	2625 (16.76)	0.03
Diabetes	61 (0.39)	164 (0.11)	0.06	57 (0.36)	58 (0.37)	0.01
Renal disease	194 (1.24)	344 (0.23)	0.12	188 (1.20)	157 (1.00)	0.02
Liver disease	532 (3.39)	1273 (0.86)	0.18	522 (3.33)	455 (2.90)	0.02
Inflammatory bowel disease	70 (0.45)	181 (0.12)	0.06	67 (0.43)	58 (0.37)	0.01
Systemic prescription drug use						
Corticosteroid	4362 (27.77)	32 001 (21.65)	0.14	4339 (27.70)	4372 (27.91)	0.00
Retinoid	69 (0.44)	57 (0.04)	0.08	66 (0.42)	49 (0.31)	0.02
Aminoglycoside	3344 (21.29)	13 220 (8.94)	0.35	3328 (21.24)	3266 (20.85)	0.00
Proton pump inhibitor	67 (0.43)	240 (0.16)	0.05	65 (0.41)	43 (0.27)	0.01
Tetracycline	330 (2.10)	379 (0.26)	0.17	325 (2.07)	252 (1.61)	0.03

FQ: fluoroquinolone; SD: standard deviation.

Data are expressed as percentages unless indicated otherwise.

^a^At the first prescription of study drugs (fluoroquinolones and amoxicillin).

### Risk of musculoskeletal AEs

There were 307 incident AEs (1.95%) in the FQ-treated cohort compared to 1,075 (0.73%) in the amoxicillin-treated group. Of the 307 cases observed after FQ treatment, 177 (57.65%) were males, and the mean age was 14 ± 2 years. Table 2 includes the hazard ratios (HRs) of musculoskeletal AEs associated with FQs and covariates. Pediatric FQ use was associated with an increased risk of musculoskeletal disorders (crude HR, 2.82; 95% confidence interval (CI), 2.48–3.20; *p* < 0.0001). The risk of musculoskeletal diseases after FQ exposure decreased after multivariable adjustment but remained statistically significant (adjusted HR, 1.18; 95% CI, 1.02–1.36; *p* = 0.022). After propensity score matching, the statistical significance of the HRs) for musculoskeletal diseases was also maintained (adjusted HR, 1.19; 95% CI, 1.01–1.40; *p* = 0.042). The unadjusted association was stronger in males and older children as well as patients with a history of diabetes, renal disease, liver disease, systemic corticosteroid use, aminoglycoside use, and tetracycline use before propensity score matching. The multivariable Cox proportional hazards model in propensity score-matched cohorts revealed that the male gender (adjusted HR, 1.39; 95% CI, 1.17–1.64; *p* = 0.0001) and older age (per year, adjusted HR, 1.11; 95% CI, 1.08–1.15; *p* < 0.0001) were only significantly associated with musculoskeletal AEs.

**Table 2:** Results of the Cox model estimating hazard ratios of musculoskeletal adverse events associated with fluoroquinolone use and other risk factors when compared with that associated with amoxicillin use in pediatric patients.

Patient characteristics	Crude HR	95% CI	P-value	Adjusted HR^a^	95% CI	P-value	Adjusted HR^a^ in PS-matched cohort	95% CI	P-value
Fluoroquinolone use	2.82	2.48–3.20	<0.0001	1.18	1.02–1.36	0.022	1.19	1.01–1.40	0.042
Male gender, vs female	1.15	1.03–1.27	0.012	1.16	1.05–1.29	0.005	1.39	1.17–1.64	0.0001
Age^b^, per year	1.11	1.10–1.12	<0.0001	1.10	1.09–1.11	<0.0001	1.11	1.08–1.15	<0.0001
Comorbidities									
Asthma	1.05	0.91–1.21	0.496	—	—	—	—	—	—
Diabetes	2.70	1.12–6.50	0.027	1.58	0.66–3.82	0.308	1.23	0.39–3.84	0.726
Renal disease	2.47	1.37–4.47	0.003	1.27	0.70–2.31	0.433	1.38	0.71–2.68	0.338
Liver disease	1.95	1.35–2.82	0.0004	1.11	0.77–1.62	0.571	1.09	0.71–1.67	0.700
Inflammatory bowel disease	1.89	0.71–5.05	0.202	—	—	—	—	—	—
Systemic prescription drug use									
Corticosteroid	1.20	1.07–1.36	0.003	1.08	0.96–1.22	0.198	1.02	0.85–1.23	0.812
Retinoid	2.88	0.93–8.93	0.067	0.99	0.32–3.10	0.988	0.71	0.18–2.85	0.626
Aminoglycoside	1.49	1.29–1.74	<0.0001	1.00	0.86–1.17	0.969	1.17	0.96–1.41	0.112
Proton pump inhibitors	1.94	0.81–4.68	0.138	1.29	0.54–3.12	0.566	0.42	0.06–2.98	0.385
Tetracycline	3.45	2.22–5.37	<0.0001	1.39	0.89–2.17	0.152	1.26	0.77–2.09	0.360

CI: confidence interval; HR: hazard ratio; PS: propensity score.

^a^Adjusted for variables for which p-values of univariable tests <0.2 (sex, age, diabetes, liver disease, renal disease, prescription of corticosteroids, retinoid, aminoglycoside, proton pump inhibitors, or tetracycline).

^b^At the first prescription of study drugs (fluoroquinolones and amoxicillin).

Analyses stratified by age and subtype of musculoskeletal outcomes revealed that the hazards of musculoskeletal AEs following FQ treatment, when compared with those associated with amoxicillin use, differed between stratified groups **(**Table 3**)**. Only children older than 10 years showed a significantly increased hazard of FQ-associated musculoskeletal AEs (adjusted HR, 1.22; 95% CI, 1.03–1.45; *p* = 0.022). Adjusted risks stratified by outcome subtype were statistically non-significant because of the small number of events after stratification. However, the risk of soft tissue disorders such as tendon injuries was marginally significant (adjusted HR, 1.20; 95% CI, 0.97–1.49; *p* = 0.095). However, the follow-up periods was extended to 90 or 180 days, the risk became non-significant (Table 3). Among the various types of FQs, only ciprofloxacin (adjusted HR, 2.03; 95% CI, 1.29–3.21; *p* = 0.002) and levofloxacin (adjusted HR, 2.08; 95% CI, 1.14–3.81; *p* = 0.018) significantly increased the risk of musculoskeletal disorders when compared to the incidence associated with amoxicillin treatment **(**Table 4**)**.

**Table 3:** Risk of musculoskeletal adverse events associated with fluoroquinolone use when compared with that associated with amoxicillin use according to age, subtype of adverse events, and duration of the follow-up period.

	Propensity score-matched cohort					
	Fluoroquinolone (n = 15 665)	Amoxicillin (n = 15 665)	Crude HR (95% CI)	P value	Adjusted HR^a^ (95% CI)	p-value
	No. of events	No. of events				
**Primary analysis (60-day follow-up period)**	305	269	1.18 (1.00–1.39)	0.045	1.19 (1.01–1.40)	0.042
**Age**^**b**^ **(years)**						
≤5	0	1	—	—	—	—
6–10	24	28	0.89 (0.51–1.53)	0.662	0.90 (0.52–1.56)	0.710
11–17	281	240	1.22 (1.03–1.45)	0.024	1.22 (1.03–1.45)	0.022
**Subtype of MAEs**						
Arthropathy	66	61	1.13 (0.80–1.60)	0.494	1.14 (0.80–1.61)	0.464
Dorsopathy	55	50	1.15 (0.78–1.68)	0.480	1.16 (0.79–1.70)	0.448
Soft tissue disorder	179	155	1.20 (0.97–1.49)	0.091	1.20 (0.97–1.49)	0.095
Osteopathy/chondropathy	13	10	1.36 (0.60–3.11)	0.463	1.39 (0.61–3.16)	0.438
**Duration of the follow-up period**						
90 days	477	403	1.11 (0.97–1.27)	0.122	1.12 (0.98–1.28)	0.094
180 days	948	807	1.06 (0.96–1.16)	0.256	1.07 (0.97–1.17)	0.182

CI: confidence interval; HR: hazard ratio; MAE: musculoskeletal adverse event.

^a^Adjusted for sex, age, diabetes, liver disease, renal disease, prescription of corticosteroids, retinoid, aminoglycoside, proton pump inhibitors, or tetracycline.

^b^At the first prescription of study drugs (fluoroquinolones and amoxicillin).

**Table 4:** Risk of musculoskeletal adverse events associated with specific fluoroquinolone use when compared with that associated with amoxicillin.

	Propensity score-matched cohort					
	Fluoroquinolone	Amoxicillin	Crude HR (95% CI)	p-value	Adjusted HR^a^ (95% CI)	p-value
	Events/patients	Events/patients				
**Primary analysis (All FQs)**	305/15 665	269/15 665	1.18 (1.00–1.39)	0.045	1.19 (1.01–1.40)	0.042
**Type of FQs**						
Ciprofloxacin	54/2077	28/2077	2.02 (1.28–3.19)	0.003	2.03 (1.29–3.21)	0.002
Levofloxacin	31/1493	16/1493	2.05 (1.12–3.74)	0.020	2.08 (1.14–3.81)	0.018
Ofloxacin	182/10 169	164/10 169	1.16 (0.94–1.43)	0.175	1.16 (0.94–1.43)	0.168
Norfloxacin	31/1768	25/1768	1.29 (0.76–2.19)	0.339	1.30 (0.77–2.20)	0.330

CI: confidence interval; FQ: fluoroquinolone; HR: hazard ratio.

^a^Adjusted for sex, age, diabetes, liver disease, renal disease, prescription of corticosteroid, retinoid, aminoglycoside, proton pump inhibitors, or tetracycline.

^b^At the first prescription of study drugs (fluoroquinolones and amoxicillin).

## Discussion

This population-based, longitudinal, observational cohort study, investigated the association between pediatric FQ use and musculoskeletal AEs and compared it with that of amoxicillin use. Despite the many concerns associated with musculoskeletal safety after FQ use in children considering the usefulness of pediatric FQs in some cases, very few studies have examined the risk of musculoskeletal disorders associated with pediatric FQ exposure and its risk factor. To the best of the authors’ knowledge, this is the first study to investigate the potential risk factors that may affect the association between pediatric FQ use and musculoskeletal AEs using a nationwide longitudinal database. This study used the large-scale nationwide health insurance claims database of South Korea to investigate pediatric FQ use in real clinical practice because safety concerns regarding pediatric FQ use limit prospective clinical trials.

This study demonstrates that pediatric FQ use is associated with a risk of musculoskeletal AEs, which is in line with common concerns. The most concerning musculoskeletal AE involved soft tissue disorders, which showed a borderline significant association. These findings differ from those of a previous cohort study that used a similar study design to the one used here, which suggests that the risk of tendon or joint disorders after FQ use is comparable with that of azithromycin^10^. Although this previous study^10^ included approximately 6,000 FQ-treated children, this study included more than 15,000 participants in the FQ cohort, making it more likely to detect differences in the incidence of rare AEs between the FQ and amoxicillin cohorts. Additionally, amoxicillin was selected as a comparator drug instead of azithromycin, which is infrequently used in Korean pediatric patients because of a high rate of resistance in Asia^17^. Amoxicillin was used in this study because: (1) it is commonly prescribed to pediatric patients in South Korea, and (2) it has not been associated with musculoskeletal AEs^12^.

Very few studies have estimated the hazards of FQ-associated musculoskeletal diseases and their risk factors in children. In adult populations, several risk factors for FQ-associated tendinopathy such as increased age, the male gender, glucocorticoid use, diabetes, and renal failure have been reported^18–22^. The male gender and increased age were associated with an elevated risk in pediatric patients, which agrees with findings from previous studies involving adults^19,20^. However, steroid use, diabetes, and renal disease did not significantly affect the incidence of musculoskeletal AEs after matching and adjustments. However, the small number of patients with a history of diabetes or renal disease may make it difficult to detect differences in the incidence of AEs between the two groups. Steroid use is often considered to be a risk factor for FQ-induced tendinopathy in adults^19–23^, but it is unclear whether this observation applies to children as well. The non-significant association between steroid use and the occurrence of side effects observed in the current study may be associated with differences in patterns of steroid use and pharmacokinetic/pharmacodynamic characteristics between children and adults. The differences in the clinical features of AEs and patterns of drug use between children and adults should be clarified in further studies.

Cox regression analyses stratified by age and subtype of musculoskeletal AEs were conducted. Of the three age groups (≤5, 6–10, and 11–17 years), only pediatric patients over 10 years of age showed a significantly increased risk. A previous review of case reports revealed that the age range for increased incidence of FQ-associated musculoskeletal diseases was 14–17 years^2^. A systematic review pooling various studies reported that the median age of occurrence was 10 years^9^. Findings from the current study and previous literature suggest that adolescents who usually have a high level of physical activity may be more vulnerable to FQ-induced musculoskeletal AEs.

The results of this study suggest that among the different subtypes of musculoskeletal AEs, soft tissue disorders such as tendinopathy may be a concern in children as well as in adults. Several studies have demonstrated the association between FQ use and soft tissue disorders such as tendinitis and tendon rupture in adults^18,23,24^. Based on prior findings, the US FDA issued a black box warning regarding tendon injuries after FQ use in 2008 and added warning and caution notices on medication labels^25^. According to case reports^4,5^, manifestations of musculoskeletal AEs following FQ treatment in children or adolescents are known to include arthropathies such as joint pain, tendon disorders, and myalgia. Although arthropathy and chondropathy are often referred to as FQ-related musculoskeletal disorders in children, the findings of this study indicate that healthcare professionals should also be cautious of tendon disorders when prescribing FQs to pediatric patients. Ciprofloxacin and levofloxacin were approved for use in children despite safety concerns because of rapidly increasing levels of resistance to other antibiotics and the limited availability of pediatric antibiotics^26^. In this study, however, ciprofloxacin and levofloxacin were significantly associated with AEs. The scarce data available^1,27,28^ indicate that pharmacokinetic and pharmacodynamic characteristics vary across FQ compounds, but there is very little theoretical or experimental evidence to explain the different risks observed among FQs. Until further studies are conducted, careful monitoring of musculoskeletal AEs is necessary when ciprofloxacin and levofloxacin are used.

However, the risk of AEs decreased gradually after increasing the follow-up period to 90 and 180 days. This coincides with other studies that reported a relatively rapid onset of musculoskeletal disease following FQ treatment^2,6,9^. The current study provides further evidence regarding the clinical patterns of musculoskeletal toxicity following FQ treatment in pediatric patients.

The mechanisms through which FQs cause musculoskeletal toxicity in children have not been identified, although several have been proposed. FQs appear to cause collagen toxicity by inhibiting synthesis or leading to oxidative damage^29^. Other experimental studies suggest that an FQ-induced magnesium deficiency may affect chondrocyte function via reduced integrin-mediated signal transduction^30,31^. The current study provides additional evidence that connects experimental and clinical data regarding pediatric musculoskeletal toxicity after FQ use.

The results presented here should be carefully interpreted. National claims data on an individual level do not offer information such as isolated pathogens, or antimicrobial susceptibility. The database covers one million participants, but the information on rare cases might be insufficient. Initially, we considered cancer (ICD 10 code C00-C97) as a meaningful comorbidity. However, in our final FQ pediatric cohorts, the proportion of patients with cancer as a comorbidity was fairly limited to present meaningful data. It was also difficult to differentiate treatment vs. prophylaxis based on this database alone, which is an inherent limitation of insurance databases. However, with regard to FQ use in pediatric patients with cancer, including prophylactic use, future studies should consider cancer as one of the comorbidities. Though diagnostic codes according to the International Classification of Disease, 10th Revision (ICD-10) were collected for whole prescribing events, the question of whether pediatric FQ use can be a definitive cause of musculoskeletal disorders or with its association in other countries cannot be answered. We tried to eliminate possible confounding factors by excluding patients with known musculoskeletal disorders, excluding patients hospitalized in the previous 90 days, and using amoxicillin as a comparator drug since it has not been associated with musculoskeletal toxicity. Various methods to control for confounding factors commonly seen in observational studies were applied. As an active comparator, amoxicillin was also adapted to reduce confounding by indications and a matching method based on propensity scores derived from various covariates was applied to overcome the potential bias derived from any differences between the two treatment groups. However, it cannot be ruled out that unmeasurable confounding factors remained. In addition, the risk of musculoskeletal side effects due to FQs was higher than that due to amoxicillin, but the relative risk of FQs compared to other antibiotics remains to be elucidated. In the future, clinical trials that include an adequate risk management plan feasible for patients, parents, and drug companies are necessary to definitively determine whether FQ treatment can cause musculoskeletal AEs in pediatric patients.

Despite such limitations, the current study showed a meaningful increased risk of musculoskeletal AEs after FQ treatment when compared to amoxicillin-treated cohorts. This association was stronger in males, older patients, and ciprofloxacin or levofloxacin users. Also, since valuable prospective clinical trials designs are limited due to ethical issues or to the large number of participants necessary to observe rare FQ-induced musculoskeletal diseases, large population-based cohort studies using real-world data such as the one presented here are necessary as alternatives.

## Conclusion

This population-based, longitudinal, cohort study showed that pediatric patients exposed to systemic FQs were at a slightly increased risk for musculoskeletal AEs than those exposed to amoxicillin, especially in males, older patients, and patients exposed to some FQs. Though, FQs have been suggested as the drug of choice to treat complicated infections in children, possible occurrence of musculoskeletal AEs should be carefully monitored in groups with certain risk factors. In the future, well-designed pragmatic clinical trials are required to clearly determine the FQs safety issues in pediatric patients.

## Methods

### Data sources

This study was approved by the Institutional Review Board of Chung-Ang University (IRB No. 1041078-201807-HR-142-01). Waiver of informed consent for clinical data collection was also approved by the IRB as all data were de-identified and collected retrospectively. This retrospective, longitudinal, cohort study used the NHIS–NSC. Under the universal and mandatory health coverage system for all citizens in Korea, the NHIS is a single insurer responsible for operating the national health insurance system^32^. The NHIS developed the NHIS–NSC database using the reimbursement claim records of approximately one million participants (2% of all Korean citizens) from January 2002 to December 2015, including inpatient, outpatient, and outpatient pharmacy claims records^32^. This database includes demographic and medical information such as gender, age, socioeconomic status, details of treatment and prescriptions, and diagnostic codes according to the ICD-10. A previous study evaluated the overall rate of agreement between the recorded diagnostic codes and the validity of the Korean health insurance database^33^.

### Cohort definition and study design

The cohort consisted of pediatric patients under the age of 18 who were prescribed systemic FQs or amoxicillin, the comparator, between January 2003 and December 2014. Drugs in this study included oral and parenteral FQs available in Korea; topical FQs such as eye drops and ear drops were excluded. Amoxicillin was used as a comparator drug to control potential confounding by indication since it has not been associated with musculoskeletal toxicity^34^. Supplementary Table 1 lists the Korean national drug codes for FQ and amoxicillin.

Eligible subjects were identified based on the following inclusion criteria: patients who did not use FQs before the index date or amoxicillin within the last year, had no diagnosis of musculoskeletal disorders (ICD-10 codes of primary outcome) within the last year, and had not been admitted to a hospital in the previous 90 days (information on antibiotic exposure during hospitalization is not available).

**Figure 2:**
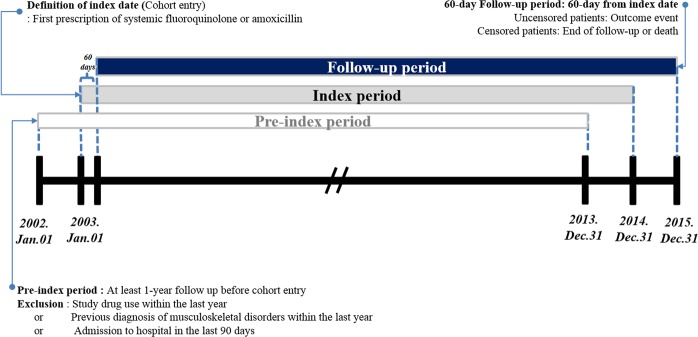
Cohort definition and study design.

Cohort entry was defined as the date of first FQ or amoxicillin prescription (index date). Patients were followed until diagnosed with the outcome event, death, prescription of other antibiotics (i.e., amoxicillin prescription in the FQ cohort and FQ prescription in the amoxicillin cohort after the index date), or the end of the follow-up period (60 days). Figure 2 outlines the study design. FQ or amoxicillin exposure was defined based on the prescription record, which is available in this cohort database. Each exposure was defined as the duration of the first prescription in the observed index period. Continuous prescriptions were allowed a 7-day refill gap.

### Primary outcome: musculoskeletal AEs

The primary outcome was the first diagnosis of adverse musculoskeletal events based on the primary and secondary diagnostic codes (ICD-10 codes). Musculoskeletal AEs included arthropathies (M02, M08-M09, and M11-M25), dorsopathies (M42-M54), soft tissue disorders such as myositis and tendon disorders (M60-M79), chondropathies and some osteopathies (M87 and M90-M94), and other musculoskeletal disorders and injuries (M95, M99, S16, S46, S56, S66, S76, S86, S96, and G72). Two investigators conducted case identification, and disagreements were resolved by consensus. Patients diagnosed with musculoskeletal system and connective tissue diseases one year prior to the index date were considered to have a history of musculoskeletal disorders and were excluded.

Outcomes were identified during a 60-day follow-up period according to the risk window used in a previous study^10^. Secondary analyses using 90-day and 180-day follow-up periods were performed to identify the high-risk time period.

### Covariates

Patient characteristics potentially associated with the occurrence of study drug prescriptions and outcome events were included to control for potential confounding. The study also included demographic characteristics such as age and sex. Medical characteristics known to be confounders, such as comorbidities and prescription drug use, were selected according to previous studies^18,19,23,24^. Comorbidities were based on diagnostic codes prior to the index date and included asthma, diabetes mellitus, renal disease, liver disease, and inflammatory bowel disease. Prescription drugs used in in- and outpatient settings within one year prior to the index date were identified using the first four digits of the Korean national drug codes. Prescription drugs were selected according to criteria established by previous studies^18,23^ and by a list of drugs with a substantial number of musculoskeletal AE reports^35^. Drugs such as systemic corticosteroids, retinoids, aminoglycosides, proton pump inhibitors, and tetracyclines were included. Supplementary Table 2 lists the codes for comorbidities and prescription drugs.

### Statistical analysis

The FQ and amoxicillin cohorts were selected based on the inclusion and exclusion criteria provided, and the baseline characteristics of patients in both cohorts were recorded. Categorical variables were compared using chi-squared tests and continuous variables were compared using independent t-tests.

To adjust for potential confounders associated with the use of antibiotics and study outcome, the FQ and amoxicillin cohorts were matched in a 1:1 ratio using the propensity score method. A logistic regression model that included the covariates mentioned above estimated propensity scores for the treatment probabilities. The greedy 5 → 1 digit matching algorithm was used^36^. Standardized difference assessed the balance after matching. Standardized differences of less than 0.1 indicated that both cohorts were well-balanced.

Cox proportional hazard regression models were used to estimate the risk of musculoskeletal AEs associated with FQ and amoxicillin use. Plotting martingale residuals against time confirmed that the proportional hazards assumption was satisfied^37^. Before the multivariate analyses were conducted, comorbidities and prescription drug use, which were selected as covariates, were tested via univariate analysis. Only those with p-values of less than 0.2 were included in the multivariate models. Estimated risks were expressed as HRs with 95% CI. Analyses were stratified by age and subtype of musculoskeletal outcomes. Furthermore, FQs were investigated to determine which one showed the strongest association with musculoskeletal AEs, and the risk within various follow-up periods (90-day and 180-day) was calculated.

SAS software (v9.4, SAS Institute, Cary, NC, USA) was used for all statistical analyses. Categorical data are expressed as percentages and continuous data as means ± standard deviation (SD). All tests were two-sided, with p-values < 0.05 and 95% CIs non-overlapping 1.0 considered to be statistically significant.

### Ethical approval

This study was approved by the Institutional Review Board of Chung-Ang University (IRB No. 1041078-201807-HR-142-01). Informed consent was not required as all data were de-identified and collected retrospectively.

## Supplementary information

Supplementary Information

## Data Availability

The data that support the findings of this study are available from the National Health Insurance Service but restrictions apply to the availability of these data, which were used under license for the current study, and so are not publicly available. Data are however available from the authors upon reasonable request and with permission of the National Health Insurance Service.
