# Supplementary material for: Real-world safety evaluation of musculoskeletal adverse events associated with Korean pediatric fluoroquinolone use: a nationwide longitudinal retrospective cohort study
Source: Sci Rep. 2019 Dec 27;9:20156. doi: 10.1038/s41598-019-56815-y (PMC6934562; doi:10.1038/s41598-019-56815-y)
Supplement: Supplementary file 1 — Supplementary Information [file 41598_2019_56815_MOESM1_ESM.pdf]

Real-world safety evaluation of musculoskeletal adverse events associated with Korean pediatric fluoroquinolone use: a nationwide longitudinal retrospective cohort study

Yoonhye Kim[1#], Minwoo Paik[1#], Chan Joo Khan[2], Yae Jean Kim[3], EunYoung Kim[1,2*]

[1]Clinical Data Analysis, Evidence based clinical research Lab., Departments of Health Science & Clinical Pharmacy, College of Pharmacy, Chung-Ang University, Seoul 06974, Republic of Korea

[2]Department of Pharmaceutical Industry, Chung-Ang University, Seoul 06974, Republic of Korea

[3]Department of Paediatrics, Samsung Medical Centre, Sungkyunkwan University School of Medicine, Seoul 06351, Republic of Korea

Supplementary Table 1. Korean national drug codes[a] for study drugs (fluoroquinolones and amoxicillin)

Drug name	ATC code	Korean national code digits 1–4	Drug name	ATC code	Korean national code digits 1–4
Ciprofloxacin	J01MA02	1341	Sparfloxacin	J01MA09	2307
Enoxacin	J01MA04	1520	Tosufloxacin	-	2422
Fleroxacin	J01MA08	1594	Rufloxacin	J01MA10	3588
Levofloxacin	J01MA12	1832	Moxifloxacin	J01MA14	3803
Lomefloxacin	J01MA07	1849	Balofloxacin	-	4289
Norfloxacin	J01MA06	2033	Gatifloxacin	J01MA16	4348
Ofloxacin	J01MA01	2039	Gemifloxacin	J01MA15	4429
Pefloxacin	J01MA03	2095			
**Drug name**	**ATC code**	**Korean national code digits 1–4**	**Drug name**	**ATC code**	**Korean national code digits 1–4**
Amoxicillin	J01CA04	1081, 1082	Amoxicillin + clavulanate	J01CR02	3104, 3105, 3106, 3107, 3585, 4401, 4465, 4620, 4672, 4673, 4674, 4676, 5040, 5041, 3289, 3290
			Amoxicillin + sulbactam	J01CR02	3798, 3799, 3800, 3813, 3814, 3815

ATC: Anatomical therapeutic chemical.

[a]Korean national drug codes consist of 9 digits (digit 1–4: main ingredients; 5–6: single or combination ingredient; 7: administration route; 8–9: dosage form).

Supplementary Table 2. Covariates included in the propensity score and multivariable Cox model with International Classification of Disease, 10th Revision (ICD-10) and Korean national drug codes

Covariates	Code
Comorbidities	ICD-10
Asthma	J45, J46
Diabetes mellitus	E10, E11, E12, E13, E14, P702
Renal disease	N00–N08, N17–N19, N25, I12, I13, Z49
Liver disease	B18, I85, I982, I983, K71–K77
Inflammatory bowel disease	K50, K51
Prescription drug use	Korean national code digit 1–4
Corticosteroid	1602, 1164, 1165, 2969, 1419, 1420, 1422, 1933, 1935, 1936, 2170, 2173, 2432, 2433, 1709, 1712, 1408, 1145, 1194
Retinoid	1787, 1023, 2430
Aminoglycoside	2321, 2409, 3288, 1651, 1794, 1068, 2007, 2279, 2238, 1777, 3609
Proton pump inhibitor	2044, 2045, 2088, 2089, 1813, 2222, 3672, 4980, 5099, 4594, 5055, 4886
Tetracycline	1497, 1495, 2074, 2367, 1959, 4953
